# Secure and Fair Cluster Head Selection Protocol for Enhancing Security in Mobile Ad Hoc Networks

**DOI:** 10.1155/2014/608984

**Published:** 2014-03-27

**Authors:** B. Paramasivan, M. Kaliappan

**Affiliations:** ^1^Department of Computer Science and Engineering, National Engineering College, Kovilpatti, Tamil Nadu 628503, India; ^2^Department of Information Technology, National Engineering College, Kovilpatti, Tamil Nadu 628503, India

## Abstract

Mobile ad hoc networks (MANETs) are wireless networks consisting of number of autonomous mobile devices temporarily interconnected into a network by wireless media. MANETs become one of the most prevalent areas of research in the recent years. Resource limitations, energy efficiency, scalability, and security are the great challenging issues in MANETs. Due to its deployment nature, MANETs are more vulnerable to malicious attack. The secure routing protocols perform very basic security related functions which are not sufficient to protect the network. In this paper, a secure and fair cluster head selection protocol (SFCP) is proposed which integrates security factors into the clustering approach for achieving attacker identification and classification. Byzantine agreement based cooperative technique is used for attacker identification and classification to make the network more attack resistant. SFCP used to solve this issue by making the nodes that are totally surrounded by malicious neighbors adjust dynamically their belief and disbelief thresholds. The proposed protocol selects the secure and energy efficient cluster head which acts as a local detector without imposing overhead to the clustering performance. SFCP is simulated in network simulator 2 and compared with two protocols including AODV and CBRP.

## 1. Introduction

MANETs are formed arbitrarily by a set of mobile devices falling within the transmission range of each other [1]. Routing protocols act as binding force in MANETs and facilitate communication beyond the physical wireless range of the nodes. In MANETs, every node cooperates with other nodes for forwarding packets to their destination by acting as a router. These protocols could operate in either flat or hierarchical network architecture. In the flat architecture, all nodes participate in the routing process. In the hierarchical architecture nodes are divided into a number of clusters each of which is managed by a cluster head that makes control decisions for cluster members. In this architecture, only cluster heads and gateway nodes are participating in the routing. Traditional MANETs routing protocols have no predefined trust exists between communication partners. This may render the network vulnerable to malicious attacks. Selfish nodes do not propagate packets from other nodes, while malicious nodes may perform modification and impersonation attacks against the network traffic. Clustering schemes [2] organized the network into one hop disjoint clusters then elect the most qualified and trustworthy nodes which played the role of cluster heads. Cluster heads are responsible for monitoring all the routing activities within the cluster itself.

Yu and Chong [3] and Bechler et al. [4] have proposed the survey about various clustering schemes for mobile ad hoc networks. In their paper, typical clustering schemes of MANETs are classified into six categories. In Ds based clustering, a set of dominating nodes act as cluster heads to relay routing information and data packets, such a set of nodes are called a dominating set (DS). A DS is called a connected DS (CDS) if all the dominating nodes are directly connected with each other. Low maintenance clustering protocol aimed for providing stable cluster architecture by reducing the reaffiliation rate and minimizing the reclustering situations which improve the network life time. It causes more communication overhead. The mobility aware clustering provides the cluster architecture based on mobility behavior of nodes. The idea is by grouping mobile nodes with similar speed into the same cluster; the intracluster links become tightly connected. In this approach, the reaffiliation and reclustering rate could be naturally decreased. MOBIC proposed an aggregate local mobility metric for cluster formation in which mobile nodes with low speed relative to their neighbors have more chance to become cluster heads. The energy efficient clustering approach has achieved the less energy consumption among mobile nodes that is also avoiding the node failure. In load balancing clustering approach, an optimum number of nodes are used to form the clusters. It set upper and lower limits on the number of mobile nodes that a cluster can deal with. Reclustering procedures are invoked for cluster maintenance that adjusts the number of nodes in that cluster. The combined metrics based clustering considered number of metrics to cluster configuration including node degree, residual energy capacity, and speed. This category aimed to elect most suitable cluster head in a local area and does not give preference to mobile nodes with certain attributes such as lowest ID or highest node degree. Advantage of this clustering scheme is flexibly adjusting the weighting factors for each metric for different scenarios. In this survey, it is not guaranteed that any one of them is the best for all situations.

Several trust models [5–8] have been proposed for self-organizing networks in distributed paradigm. Jiang and Baras [9] examined the efficiency of trust based reactive routing protocols in the presence of attacks in the networks. This method is considered first-hand information to evaluate other node's trust values to make trustworthiness. Yan et al. [10] proposed a secure AODV based routing protocol for an ad hoc network which is established a secure end-to-end route. The trust values are calculated based on direct observation which is transitive. Pirzada and McDonald [11] enhanced the trust management by considering the confidence level of trust of each node. They have used confidence level as a weight to compute trust value. Ghosh et al. [12] developed a trust model to strengthen the security of MANETs and they dealt with the issues associated with recommendations. Their model was utilized only trusted routes for making effective communication and isolates the malicious nodes based on the evidence obtained from direct interactions and recommendations. Ghosh et al. [13] proposed a mechanism for distinguishing selfish peers from cooperative nodes that is based on local monitoring. In order to distinguish between selfish and cooperative peers, a series of well-known statistical tests are applied for obtaining features from the observed AODV actions.

Noman Mohammed et al [9] proposed a Mechanism design based secure leader election model for encouraging mobile nodes to honestly participate in the election process in order to avoid activities of selfish nodes and balance the energy consumption among all nodes for increasing lifetime of MANETs. The objective of mechanism design [4] is to address problem of designing incentives for nodes to provide truthful information and computing optimal system wide solution for finding the optimal cost efficient leaders. Vickrey, Clarke, and Groves (VCG) model is applied for node incentives to ensure truth telling to be the dominant strategy for any node. They have proposed local election algorithms, namely, cluster-dependent leader election and cluster independent leader election which provided globally optimal election solutions with a low cost. The Nodes with the most remaining energy are elected as the cluster head. This approach makes storage overhead because the cluster head kept an extra service table and each node maintains a reputation table and neighboring nodes list.

Milan et al. proposed a scheme [14], where a game theoretic model is applied to study the impact of collisions on a hop-by-hop reputation based mechanism for regular networks with uniform random traffic. The nodes in MANETs are equipped with different resources and provide discrete services. It did not deal with irregular topologies and nonuniform routing. It also discussed the perception and interaction asymmetries that could impair cooperation between nodes.

Safa et al. presented a cluster based trust aware routing protocol (CBTRP) [15] to ensure secure routing path and established the trust based environment. This mechanism is used to distinguish the trusted nodes from malicious nodes. CBTRP makes use of the weighted clustering algorithm (WCA) [16] to elect cluster heads. The weighted degrees are taken into consideration such as battery power, number of neighbors, transmission power, and mobility of the nodes to form optimal cluster head. CBTRP has also taken security into account to form trusted clusters. It organized the network into 1-hop clusters in which every node is able to elect the most qualified and trustworthy node to be its cluster head. Cluster members forward the packets through the trusted cluster heads. Malicious nodes do not forward the packets to them. In CBTRP model, the trust value is computed based on the information that one node can gather about the other node's vital information including analyzing the received, forwarded, and overheard packets. Analyzing the node's behavior shows that the node is selfish, acting like a black hole, and carrying out a modification attack, fabrication attack and latency delays. This approach provides improved connectivity in MANETs in the presence of malicious nodes and also it ensured the passage of packets through trusted routes only by behavior of each node. Once a malicious node is discovered, it is isolated from the network such that no packet is forwarded from it.

Chatterjee et al. [17] proposed a secure trusted auction oriented clustering based routing protocol (STACRP) to provide trusted structured framework for MANETs. Two auction mechanisms, namely, procurement and Dutch, are used to determine the forwarding cost of one hop. STACRP organized the network into 1-hop clusters and elects the trustworthy nodes as cluster head (CH) by using a secret voting scheme. Each node maintains information of itself and its neighboring nodes for cluster maintenance. The trust model is analyzed using Markov chain which guarantees to selfish node to revoke its status from warned status to normal status by proper forwarding of others packets. This achieved a secure reliable routing solution. STACRP detected selfish nodes and enforces cooperation between nodes to achieve better throughput and packet delivery ratio with less routing overhead.

## 2. Materials and Methods

SFCP makes use of the weighted clustering (WCA) [18] to form 1-hop cluster in the networks. In addition to that, SFCP takes security component to form trusted CHs and also considers each node's remaining energy level to elect the cluster head (CH) in all clusters. The network has been partitioned into 1-hop disjoint clusters. It ensures the secure routing. The proposed SFCP elects the trusted CH by the mechanism called fair cluster head selection which distinguishes trusted nodes from malicious nodes. Each node elects most trustworthy node of its 1-hop neighbors to be its CH which should not be a faulty degree claim for election process. In SFCP, each node *i* takes into account its degree deg⁡_*i*_, an energy fairness factor *F*_*i*_, and a security component. Equation (1) presents the clustering score of each node *v*_*i*_

(1) $$\begin{array}{c} v_{i}=a\times \frac{\text{de}{\text{g}}_{i}}{d_{\max}}+b\times \frac{F_{i}}{F_{\max}}+c_{t}\times \left(\frac{N_{f}}{\text{de}{\text{g}}_{i}}-\frac{2}{3}\right)+d\times E, \end{array}$$

where the coefficients *a*, *b*, *c*_*t*_, and *d* satisfy the following:

(2) $$\begin{array}{c} a+b+c_{t}+d=1. \end{array}$$

deg⁡_*i*_ is the number of nodes whose Euclidean distance from *i* is less than the radio range of *i* that is, the degree of a node in a network is the number of edges the node has to other nodes. *N*_*f*_ is the ratio of the number of neighbors in their neighbors list. *F*_*i*_ defines how many times *i* has previously served as CH, *E* is the Remaining energy level of each node *i* is calculated as follows

(3) $$\begin{array}{c} E=E_{i}-E_{c}, \end{array}$$

where *E*_*i*_ is initial energy and *E*_*c*_ is consumed energy.

The secure cluster head selection algorithm describes the procedure for selecting cluster head (see Algorithm 1).

### 2.1. Byzantine Agreement

**Figure 1:**
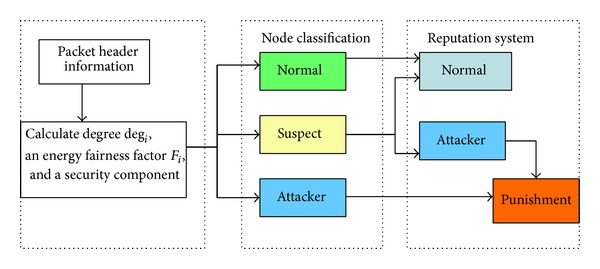
Byzantine agreement classification system.

Byzantine agreement mechanism is used to solve the problem of multiple nodes reaching agreement in the presence of malicious node and message failures. This model demands global agreement to be reached for cluster formation and malicious identification. The routing paths are selected after a mutual exchange of control message amongst the neighbouring nodes (Figure 1). The consensus problem is assumed as a set of nodes *n*. A node is deemed correct if it does not fail and it correctly follows the protocol specification. Otherwise, the node is considered to be faulty. A consensus execution is initiated when every correct node *n*_*i*_ proposes an input value *v*_*i*_ and terminates after every correct node decided on a common value *v*. Consensus is defined by three properties such as validity, agreement, and termination. In validity, if every correct node proposed the same value *v*, then any nodes can decide *v*, In agreement, no two correct nodes decide differently and termination follows after the correct node decides. Agreement ensures the consistency in which some nodes decide a value *v*, and then no other node can decide a different value. Termination ensures that nodes must decide. These properties can be broken down into two different categories which include safety and liveness. Safety restricts the bad things that can happen in the system. Liveness ensures that good things eventually happen. Both the validity and agreement are safety properties while termination belongs to liveness property.

A fault occurs in a network when a node or link deviates from its correct behaviour. Within this context, faults are classified in two main classes: omissive faults and Byzantine faults. A fault is of omissive class if there is crash of a node and a message loss in a communication link. A fault is of Byzantine faults class if there is a compromised node that acts as malicious which sends incorrect values purposely and it influence other nodes to deviate from its properties of the protocol. More precisely, the nodes must reach an agreement that is admissible in the following sense:

- all normal nodes must agree on the same value *v*;
- in case all the normal nodes have the same initial value, then *v* must be equal to this value.

It considered the following assumption that there are *n* nodes in the network in which 30% of nodes are considered as malicious. They make communication through messages. The malicious node can forge messages, send conflicting messages, and masquerade as other nodes. If a message from a normal node is lost or damaged, then the normal node is treated as attacker.

It classifies each node as normal, suspect, or attacker and allocates a different value of *c*_*t*_ for each type of node according to the byzantine agreement requirement to (4) as shown below

(4) ct={>0if  Nfdeg⁡i>23, 0.4  for  dense, 0.2  for  sparse
<0if  Nfdeg⁡i≤23=0if  deg⁡i>network  size, (a,b,d=0).

When the ratio of the search result *N*_*f*_ over deg⁡_*i*_ is less than or equal to the threshold, the node *i* is classified as suspect. A suspect node is not immediately excluded from the network, but it is penalized by reducing its score *v*_*i*_ and also this node is motivated not to claim faulty degree. The node *i* is marked as attacker; if the advertised degree deg⁡_*i*_ is equal to the network size and the threshold is exceeded, then it is immediately excluded from both the clustering and the routing procedures. The node is classified as normal if the advertised ratio of degree deg⁡_*i*_ is greater than the threshold value (see Algorithm 2).

## 3. Result and Discussion

In this section, the performances of proposed SFCP are evaluated in network simulator (NS-2) [19]. The result of SFCP provides an effective malicious free routing in the presence of attacker nodes. In this implementation models, we used the energy model and initially assigned 60 to 100 joules to each node. Besides, different numbers of nodes that vary from 50 to 150 are deployed in an area of 900 × 900 m.  Table 1 summarizes the simulation parameters. The attacker nodes were made to drop the messages during routing phase.

The following sections discussed the results and observations of the proposed SFCP along with AODV and CBRP in terms of packet delivery ratio, routing and cluster overhead, and routing latency.

### 3.1. Packet Delivery Ratio (PDR) in the Presence of Malicious Nodes

**Figure 2:**
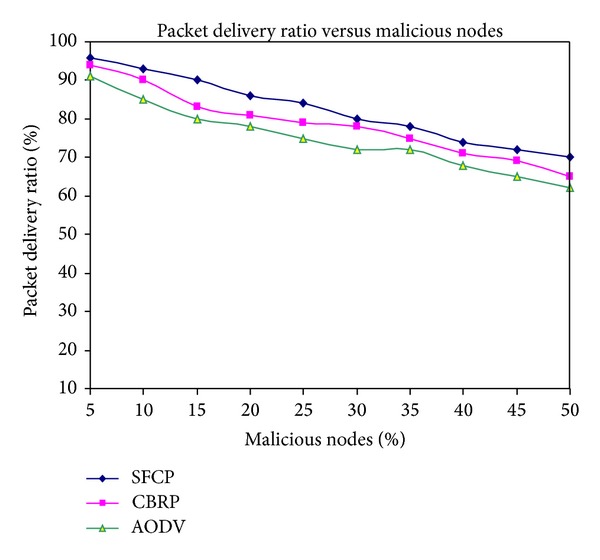
Packet delivery ratio versus malicious nodes.

In this simulation, the impacts of malicious nodes are evaluated by measuring the PDR. The numbers of nodes used in this simulation are 50 and malicious nodes are varied from 0 to 40%. In Figure 2, notice that SFCP maintained much higher PDR about 80% of the data packets when 40% of nodes are misbehaving and other protocols decreased the PDR. This is due to the fact that CBRP and AODV do not have a mechanism to detect misbehaving nodes. When numbers of malicious nodes are increased, it becomes harder to find malicious free routes from the source to the destination. SFCP could detect misbehaving or faulty claim nodes continuously changing with network mobility and also it elects misbehaving free cluster heads for routing using Byzantine Agreement node classification mechanism.

### 3.2. Routing and Clustering Overhead

**Figure 3:**
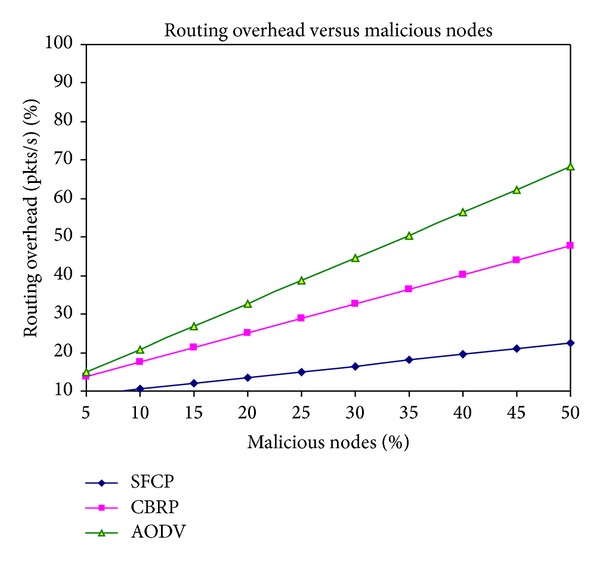
Routing overhead versus malicious nodes.

In simulation, clustering and routing overhead are analysed for SFCP, AODV, and CBRP while varying the malicious nodes. The numbers of nodes are set to 50.  Figure 3 shows that minimal routing overhead is caused by SFCP and CBRP than AODV. SFCP and CBRP are greatly reducing network traffic because of their cluster based architecture and limited exchange of routing control message. On the other hand, route request of AODV is originated from the source; then, it is flooded throughout the network until destination is reached which cause more routing overhead. In addition to that, the number of malicious node increases, and the overhead is also increased because malicious nodes drop packets. The result shows that SFCP caused minimal routing overhead when there are 40% of malicious nodes present in the network.

### 3.3. Routing Latency

**Figure 4:**
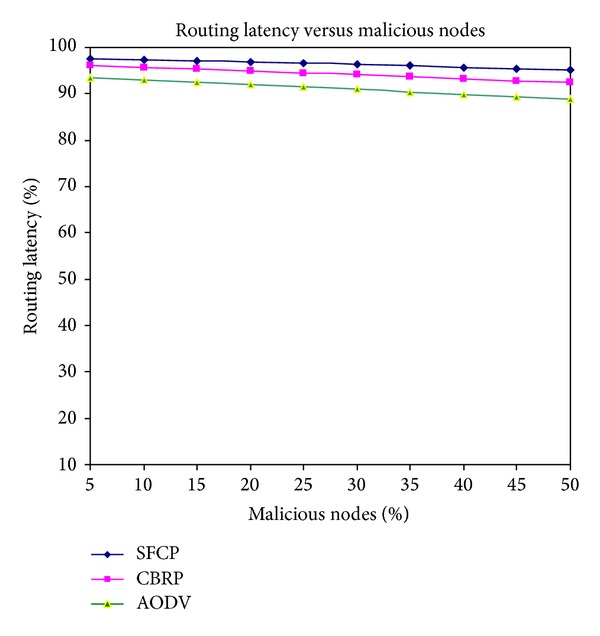
Routing latency versus malicious nodes.

Figure 4 shows that SFCP achieved higher routing latency than the other two protocols. SFCP is considered the Byzantineagreement mechanism to classify whether the node is normal or attacker. The normal nodes only allow participating in routing and cluster head selection and minimum number of nodes participated in the routing phase. Figure 4 shows that the numbers of malicious nodes are increased; routing latency is decreased for AODV and CBRP because malicious nodes drop the packets.

## 4. Conclusion

In this paper, the proposed SFCP avoided the impact of compromising nodes by selecting the secure and energy efficient node as a cluster head. SFCP can effectively classify the malicious nodes and prevent these nodes from faulty degree claim in cluster head selection. The SFCP used the Byzantineagreement mechanism to mitigate selfishness which made the network more attack resistant. SFCP could detect malicious nodes that get isolated from the networks. Simulation results show that SFCP achieved better packet delivery ratio and routing latency with less routing and cluster overhead than AODV and CBRP.

## Figures and Tables

**Algorithm 1:**
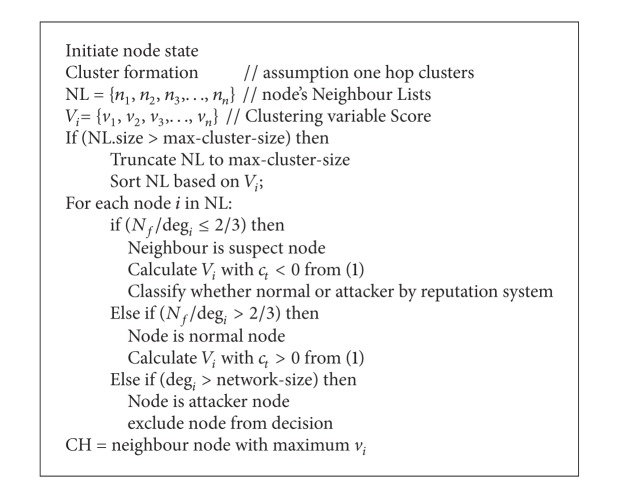
Secure cluster head selection algorithm.

**Algorithm 2:**
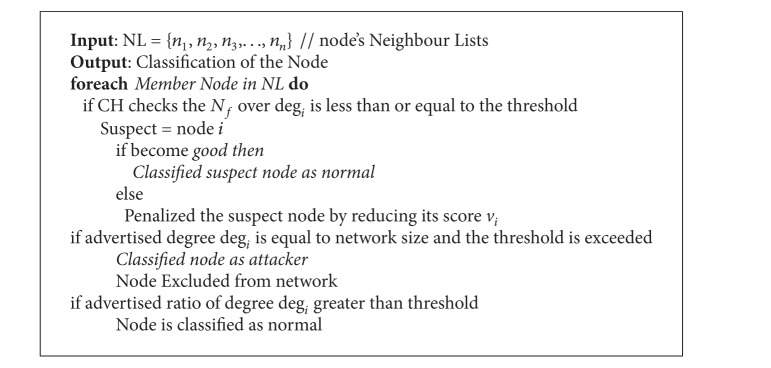
Byzantine agreement node classification algorithm.

**Table 1:** Simulation parameters.

Parameter	Value
Simulation area	900 m × 900 m
Simulation time	800 Sec
Number of nodes	50, 100, and 150
Transmission range	200 m
Movement model	Random waypoint model
Initial energy	100 joules
Packet size	512 bytes
Pause time	200 Sec
